# Microstructure evolution in amorphous Hf-B-Si-C-N high temperature resistant coatings after annealing to 1500 °C in air

**DOI:** 10.1038/s41598-019-40428-6

**Published:** 2019-03-05

**Authors:** Y. Shen, J. C. Jiang, P. Zeman, V. Šímová, J. Vlček, E. I. Meletis

**Affiliations:** 1 Department of Materials Science and Engineering, The University of Texas at Arlington, Arlington, 76019 TX USA; 2 Department of Physics and NTIS-European Centre of Excellence, University of West Bohemia, Univerzitní 8, 30614 Plzeň, Czech Republic

## Abstract

Recently, amorphous Hf-B-Si-C-N coatings found to demonstrate superior high-temperature oxidation resistance. The microstructure evolution of two coatings, Hf_7_B_23_Si_22_C_6_N_40_ and Hf_6_B_21_Si_19_C_4_N_47_, annealed to 1500 °C in air is investigated to understand their high oxidation resistance. The annealed coatings develop a two-layered structure comprising of the original as-deposited film followed by an oxidized layer. In both films, the oxidized layer possesses the same microstructure with HfO_2_ nanoparticles dispersed in an amorphous SiO_x_-based matrix. The bottom layer in the Hf_6_B_21_Si_19_C_4_N_47_ coating remains amorphous after annealing while Hf_7_B_23_Si_22_C_6_N_40_ recrystallized partially showing a nanocrystalline structure of HfB_2_ and HfN nanoparticles separated by h-Si_3_N_4_ and h-BN boundaries. The HfB_2_ and HfN nanostructures form a sandwich structure with a HfB_2_ strip being atomically coherent to HfN skins via (111)-Hf monolayers. In spite of the different bottom layer structure, the oxidized/bottom layer interface of both films was found to exhibit a similar microstructure with a fine distribution of HfO_2_ nanoparticles surrounded by SiO_2_ quartz boundaries. The high-temperature oxidation resistance of both films is attributed to the particular evolving microstructure consisting of HfO_2_ nanoparticles within a dense SiO_x_-based matrix and quartz SiO_2_ in front of the oxidized/bottom layer interface acting as a barrier for oxygen and thermal diffusion.

## Introduction

Ultra-high temperature ceramics of transition metal based borides, carbides, nitrides and their composites exhibit a combination of desirable properties including a high melting point, high hardness, superior oxidation and corrosion resistance and high thermal stability^1–14^. Recent attention to ZrB_2_-SiC and HfB_2_-SiC composites is due to their exceptional combination of high hardness and high oxidation resistance (>1000 °C), important characteristics for surface protection in extreme environments^15,16^. The HfB_2_-based materials in particular exhibit higher oxidation resistance compared to ZrB_2_-based materials and thus, possess more promise as high temperature protection coatings. Some potential applications include turbine blades and vanes, sharp wing leading edges and nose tips in hypersonic vehicles, atmospheric re-entry and rocket propulsion systems^9,17–24^.

Our previous work has been focused on Hf-B-Si-C films that were found to exhibit high hardness (up to 37 GPa), along with high electrical conductivity and high oxidation resistance in air up to 800 °C^25,26^. In view of the important role of nitrogen in the high thermal stability of Si-B-C-N films^27–31^, nitrogen was incorporated recently into the Hf-B-Si-C system to stabilize its structure^12^, limit the release of boron and extend the oxidation resistance beyond 1000 °C. The Hf-B-Si-C-N films were deposited using pulse reactive magnetron co-sputtering of a single B_4_C-Hf-Si target in argon-nitrogen gas mixtures with a negative-voltage pulse length of 85 µs^12,13,32^ or 50 µs, [^32^ this work] and a short-lived high positive voltage overshoot (higher than 200 V, as shown in^32^) after the negative voltage pulses with a repetition frequency of 10 kHz. The base pressure before the deposition was 3 × 10^−3^ Pa^12,13^ or 1 × 10^−3^ Pa, [^32^ this work]. In paper^13^, we presented a study of the high-temperature oxidation resistance mechanism of Hf_7_B_23_Si_17_C_4_N_45_ film with a contamination level <4 at.%, which was prepared at a 20% N_2_ fraction in the gas mixture and annealed in air up to 1100–1500 °C. After a reduction of the base pressure to 1 × 10^−3^ Pa, we investigated the effects of a varying 0–50% N_2_ fraction in the gas mixture on the evolution of the elemental composition, structure, mechanical, electrical and optical properties, and oxidation resistance of the films^32^. Moreover, the effects of shortened (50 µs compared to 85 µs) voltage pulses on the film properties were investigated for selected N_2_ fractions in the gas mixture. Here, it should be mentioned that the negative voltage pulses on the sputtered target need to be sufficiently short to avoid micro-arcing at the target and thus, to produce high-quality defect-free films with low surface roughness. In paper^32^, we demonstrated very high oxidation resistance in air up to 1500 °C for two sufficiently hard (20–22 GPa) Hf-B-Si-C-N films deposited at the voltage pulse length of 50 µs: the electrically conductive Hf_7_B_23_Si_22_C_6_N_40_ film prepared at the 15% N_2_ fraction in the gas mixture, and the optically transparent Hf_6_B_21_Si_19_C_4_N_47_ film prepared at the 25% N_2_ fraction in the gas mixture, both with a contamination level <3 at.%.

In this work, the Hf_7_B_23_Si_22_C_6_N_40_ and Hf_6_B_21_Si_19_C_4_N_47_ films were prepared on SiC substrates and annealed in air up to 1500 °C. Comprehensive high-resolution transmission electron microscopy (HRTEM) and selected-area electron diffraction (SAED) studies were conducted to determine the microstructures that develop during high temperature exposure in these films and to understand their superior high-temperature oxidation resistance and the important effect of nitrogen incorporation (not investigated in^13^).

This study is a part of an overall program of our laboratories at UTA and UWB conducted to develop new, sufficiently hard, thin-film materials in the Hf-B-Si-C-N system with a controlled electrical conductivity and optical transparency, and with ultrahigh thermal stability in air. When the excellent oxidation resistance at elevated temperatures of thin-film materials is combined with a high optical transparency (e.g., as in the case of Si–B–C–N films^30,31^), they can be used for high-temperature protective coatings of electronic and optical elements. Alternatively, a combination of the high oxidation resistance and electrical conductivity is desirable for harsh-environment sensors, such as capacitive pressure, vibration and tip clearance sensors (e.g., for advanced gas turbine engines^33–36^).

The pulse magnetron sputter technique used makes it possible to deposit densified films onto substrates at a floating potential (i.e., without any substrate bias) due to increased kinetic energies of sputtered target material and process gas ions at the substrates, caused by the high positive voltage overshoots after the negative voltage pulses^32^. This is of key importance for industrial applications, particularly for production of thin-film materials on large area non-conductive substrates (no rf-induced bias needed).

## Experimental

### Material preparation

The Hf_7_B_23_Si_22_C_6_N_40_ and Hf_6_B_21_Si_19_C_4_N_47_ films were synthesized in a Balzers BAS 450PM sputtering system. The films were deposited on polished and ultrasonically cleaned SiC single crystal substrates using reactive magnetron sputtering from a single B_4_C-Hf-Si target in an argon-nitrogen gas mixture. The target (127 mm × 254 mm) was prepared by positioning p-type Si and Hf stripes on a B_4_C plate (6 mm thick) with fixed 65% B_4_C + 15% Hf + 20% Si fractions in the target erosion area. A pulsed dc power supply (Rübig MP120) was used to drive the magnetron operating at a 10 kHz repetition frequency with a 500 W average target power and a fixed 50 µs negative-voltage pulse length with a short-lived high positive voltage overshoot (higher than 200 V, as shown in^32^) after the negative voltage pulses. During sputtering, the voltage pulse duration of 50 µs is sufficiently short to avoid micro-arcing at the non-conductive layer formed on the B_4_C-Hf-Si target (for details see^32^). The base pressure prior to deposition was 1 × 10^−3^ Pa. The total pressure of the gas mixture during the deposition was 0.5 Pa with a N_2_ fraction being 15% and 25% for Hf_7_B_23_Si_22_C_6_N_40_ and Hf_6_B_21_Si_19_C_4_N_47_ film, respectively. The distance from target to substrate was 100 mm and the substrate temperature was maintained at 450 °C. The substrates were held at a floating potential. These conditions produced a film thickness of 1450 nm and 1240 nm for the Hf_7_B_23_Si_22_C_6_N_40_ and Hf_6_B_21_Si_19_C_4_N_47_, respectively. Rutherford backscattering spectrometry (RBS) and elastic recoil detection (ERD) with a Van de Graaf generator with a linear electrostatic accelerator were used to determine the film composition.

The annealing (annealing denotes a high temperature exposure and oxidation in air) experiments were conducted from room temperature up to 1500 °C using a symmetrical high resolution Setaram TAG 2400 system in synthetic atmospheric air (flow rate of 1 l/h). The heating and cooling rate was 10 °C/min and 30 °C/min, respectively.

### Microstructure characterization

The crystallographic structure of the annealed films was first studied by X-ray diffraction (XRD) θ-2θ measurements utilizing a Bruker D8 Advance Diffractometer with Cu *Kα* radiation at 40 kV voltage and 40 mA current. The film microstructures were then studied by cross-section and plan-view TEM. Thin foil specimens were prepared by the procedure of mechanical grinding, polishing, and dimpling, followed by Ar-ion milling. A Hitachi H-9500 electron microscope was utilized operated at 300 keV with a point resolution of 0.18 nm.

## Results and Discussion

### XRD analysis

**Figure 1:**
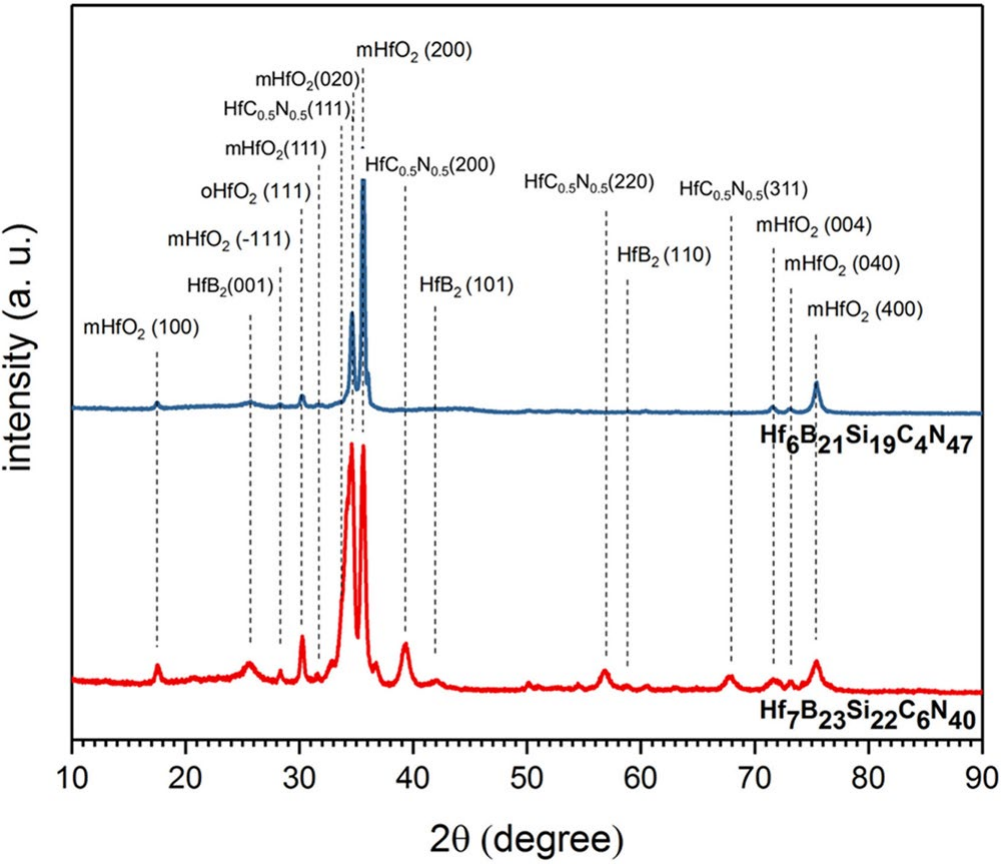
θ-2θ XRD spectra of the annealed Hf_7_B_23_Si_22_C_6_N_40_ and Hf_6_B_21_Si_19_C_4_N_47_ films.

The as-deposited and annealed films were first examined by θ-2θ XRD analysis. Both as-deposited Hf-B-Si-C-N films have a pure amorphous structure as identified by XRD and further confirmed by TEM studies (see later section). Figure 1 shows θ-2θ XRD spectra of the annealed Hf_6_B_21_Si_19_C_4_N_47_ and Hf_7_B_23_Si_22_C_6_N_40_ films. The XRD spectrum of the former annealed film shows peaks at 2θ angles of 17.55°, 28.34°, 31.60°, 34.61° and 35.64° that can be identified as monoclinic HfO_2_ (m-HfO_2_, PDF #01-075-6426, ***a = ***5.1187 Å, ***b = ***5.1693 Å, ***c = ***5.297 Å, ***β = ***99.18°, P2_1_/c)^37^ (100), ($\bar{1}$11), (111), (020) and (200), respectively. It should be noted that the latter peak is also close to orthorhombic HfO_2_ (002) (o-HfO_2_, PDF #01-070-2832, ***a = ***5.0073 Å, ***b = ***5.2276 Å, ***c = ***5.058 Å, Pbcm), and tetragonal HfO_2_ (200) (t-HfO_2_, PDF #00-008-0342, ***a = ***5.14 Å, ***c = ***5.25 Å, P4_2_/nmc). The peak located at the 2θ angle of 30.24° can correspond to (111) of the o-HfO_2_ and/or t-HfO_2_. This result is in agreement with our previous studies^12,13^ and indicates mainly formation of m-HfO_2_, o-HfO_2_ (and possibly t-HfO_2_) in the annealed Hf_6_B_21_Si_19_C_4_N_47_.

The XRD spectrum of the annealed Hf_7_B_23_Si_22_C_6_N_40_ film shows additional peaks besides those present in the spectrum of the annealed Hf_6_B_21_Si_19_C_4_N_47_ film. The peak at an angle of 39.48°, and broad peaks at angles of 56.70° and 68.00° match very well with the (200), (220) and (311) of face centered cubic (fcc) HfC_0.5_N_0.5_ (PDF#04-002-2469, a = 4.586 Å, Fm-3m), respectively. Note that the (111) HfC_0.5_N_0.5_ peak at 33.8° is on the left shoulder of the mHfO_2_ (020) peak. Also, the peaks at 25.80°, 42.03° and 58.79° match well with the (001), (101) and (110) of HfB_2_ (PDF#38-1398, hexagonal, a = 3.14 Å, c = 3.47 Å, P6/mmm)^37^. It is noted that the peak at 25.80° can also match a turbostratic structure of BN or B(C)N^38^ as reported previously^27^. Thus, in addition to HfO_2_, the annealed Hf_7_B_23_Si_22_C_6_N_40_ film showed formation of Hf(C)N solid solution, HfB_2_ and possibly BN. The XRD data and corresponding phases are summarized in Table 1.

**Table 1:** Summary of XRD data along with the type of phases formed in the annealed films.

Peak position in Hf_6_B_21_Si_19_C_4_N_47_ (2θ angle)	Crystal structure and indices						Peak position in Hf_7_B_23_Si_22_C_6_N_40_ (2θ angle)
	m-HfO_2_	o-HfO_2_	t-HfO_2_	HfC_0.5_N_0.5_	HfB_2_	BN	
17.55°	(100)						17.55°
					(001)	(002)	25.80°
28.34°	($\bar{1}$11)						28.34°
30.24°		(111)	(111)				30.24°
31.60°	(111)						31.60°
				(111)			33.80°
34.61°	(020)						34.61°
35.64°	(200)	(002)	(200)				35.64°
				(200)			39.48°
					(101)		42.03°
				(220)			56.70°
					(110)		58.79°
				(311)			68.00°
	Phases in Hf_6_B_21_Si_19_C_4_N_47_						
	Phases in Hf_7_B_23_Si_22_C_6_N_40_						

### TEM studies

#### Overall film structure

**Figure 2:**
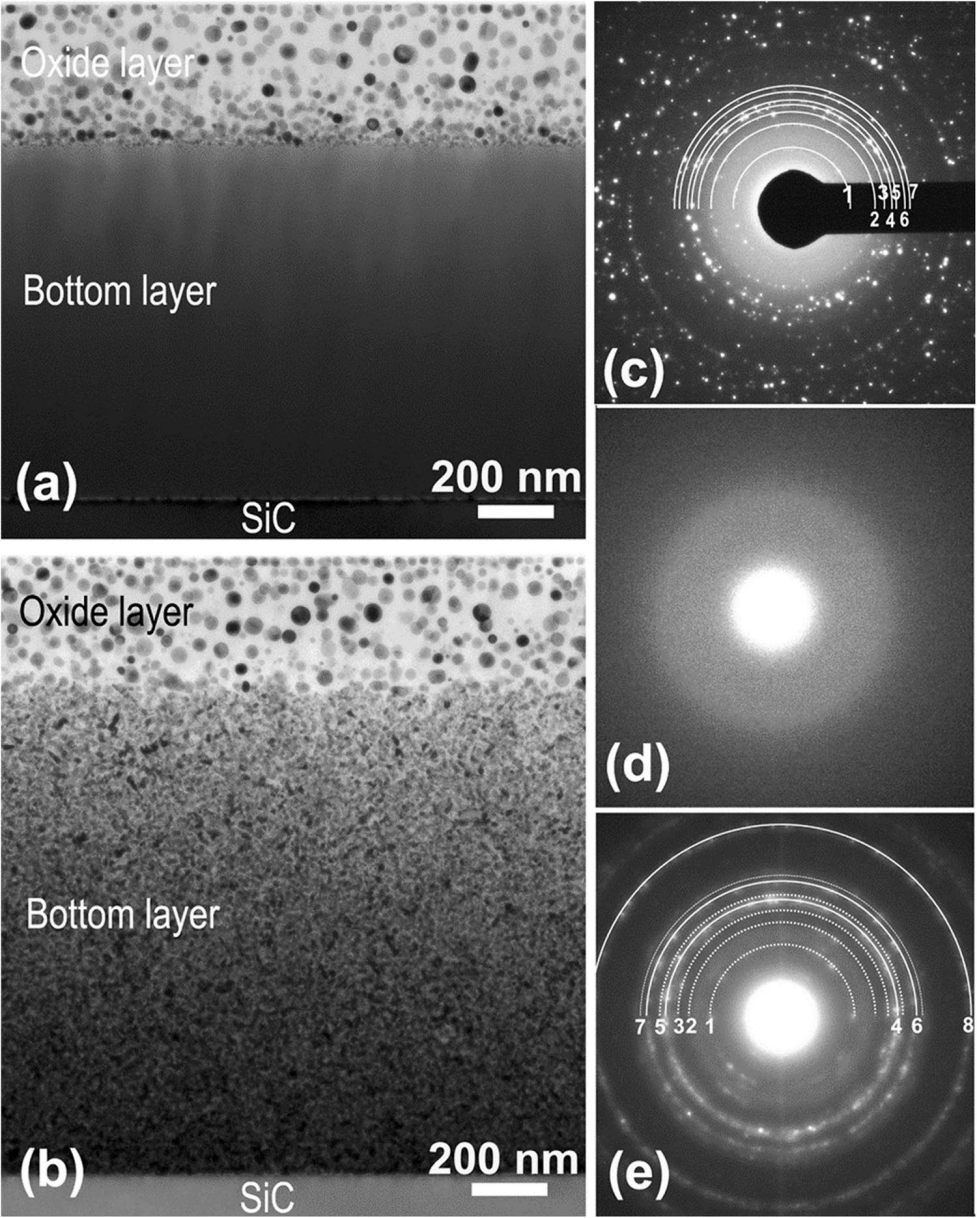
Cross-section TEM images from the annealed (**a**) Hf_6_B_21_Si_19_C_4_N_47_ and (**b**) Hf_7_B_23_Si_22_C_6_N_40_ films and SAED patterns taken from the (**c**) top nanocomposite layer, (**d**) bottom layer in the Hf_6_B_21_Si_19_C_4_N_47_ film and (**e**) bottom layer in the Hf_7_B_23_Si_22_C_6_N_40_ film.

We have employed cross-section TEM for a detailed microstructure study of the annealed films. Figure 2(a,b) presents cross-section TEM images of the annealed Hf_6_B_21_Si_19_C_4_N_47_ and Hf_7_B_23_Si_22_C_6_N_40_ films, respectively. Both annealed films have a two-layered structure: a nanocomposite layer with discrete HfO_2_ nanoparticles embedded in an amorphous SiO_x_-based matrix on the top surface followed by the remaining of the original as-deposited film at the bottom. A similar film morphology with some distinct differences however (see later section), has also been observed in the annealed Hf_7_B_23_Si_17_C_4_N_45_ film studied previously^12,13^. The top composite layer in the present annealed films has nearly the same thickness of ~360 nm and exhibits nearly the same microstructure. The entire film thickness (the total of both layers) is ~1300 nm and ~1600 nm for the Hf_6_B_21_Si_19_C_4_N_47_ and Hf_7_B_23_Si_22_C_6_N_40_ film, respectively. Figure 2(c) is a SAED pattern of the top layer in the annealed Hf_6_B_21_Si_19_C_4_N_47_ film in a plan-view TEM foil. The diffraction rings 1, 2, 3, 4, 5, 6 and 7 have a lattice spacing of 5.07 Å, 3.64 Å, 3.17 Å, 2.95 Å, 2.83 Å, 2.63 Å and 2.54 Å, respectively. The diffractions 1, 3 and 5 were uniquely identified to be the (100), ($\bar{1}$11) and (111) of m-HfO_2_, respectively. The diffraction 4 can be identified as the (111) of o-HfO_2_ or t-HfO_2_. While the diffraction rings 2, 6 and 7 can correspond to superposition of the (011)/(110) of m-HfO_2_ or (110) of o-HfO_2_, the (002) of m-HfO_2_ or (020) of o-HfO_2_ or (002) of t-HfO_2_, and the (020)/(200) of m-HfO_2_ or (002) of o-HfO_2_ or (200) of t-HfO_2_, respectively. Figure 2(c) confirms the presence of both m-HfO_2_ and o-HfO_2_ (with possible presence of t-HfO_2_) in the top layer.

The bottom layer in the annealed Hf_6_B_21_Si_19_C_4_N_47_ film exhibits a rather homogeneous amorphous structure as was further confirmed by electron diffraction, Fig. 2(d). However, the bottom layer in the annealed Hf_7_B_23_Si_22_C_6_N_40_ film exhibits a grainy structure. Figure 2(e) is a SAED pattern taken from this section of the film showing the presence of a nanocrystalline structure. The diffraction rings 1, 2, 3, 4, 5, 6, 7 and 8 have a lattice spacing of 4.30 Å, 3.35 Å, 2.92 Å, 2.64 Å, 2.53 Å, 2.29 Å, 2.20 Å and 1.62 Å, respectively. The diffraction rings 1, 2, 3 and 5 match very well with the h-Si_3_N_4_ (PDF #09-0250, ***a*** = 7.758 Å, ***c*** = 5.623 Å, P31c) (101), (200), (201) and (210), respectively. The diffraction rings 4, 6 and 8 can be identified as the (111), (200) and (220) of cubic Hf(C)N and the diffraction 7 can possibly correspond to the (101) of HfB_2_ (see comment regarding this diffraction in later section). These results clearly show that annealing caused crystallization in the original Hf_7_B_23_Si_22_C_6_N_40_ film while the amorphous structure in the annealed Hf_6_B_21_Si_19_C_4_N_47_ film was persistent.

**Figure 3:**
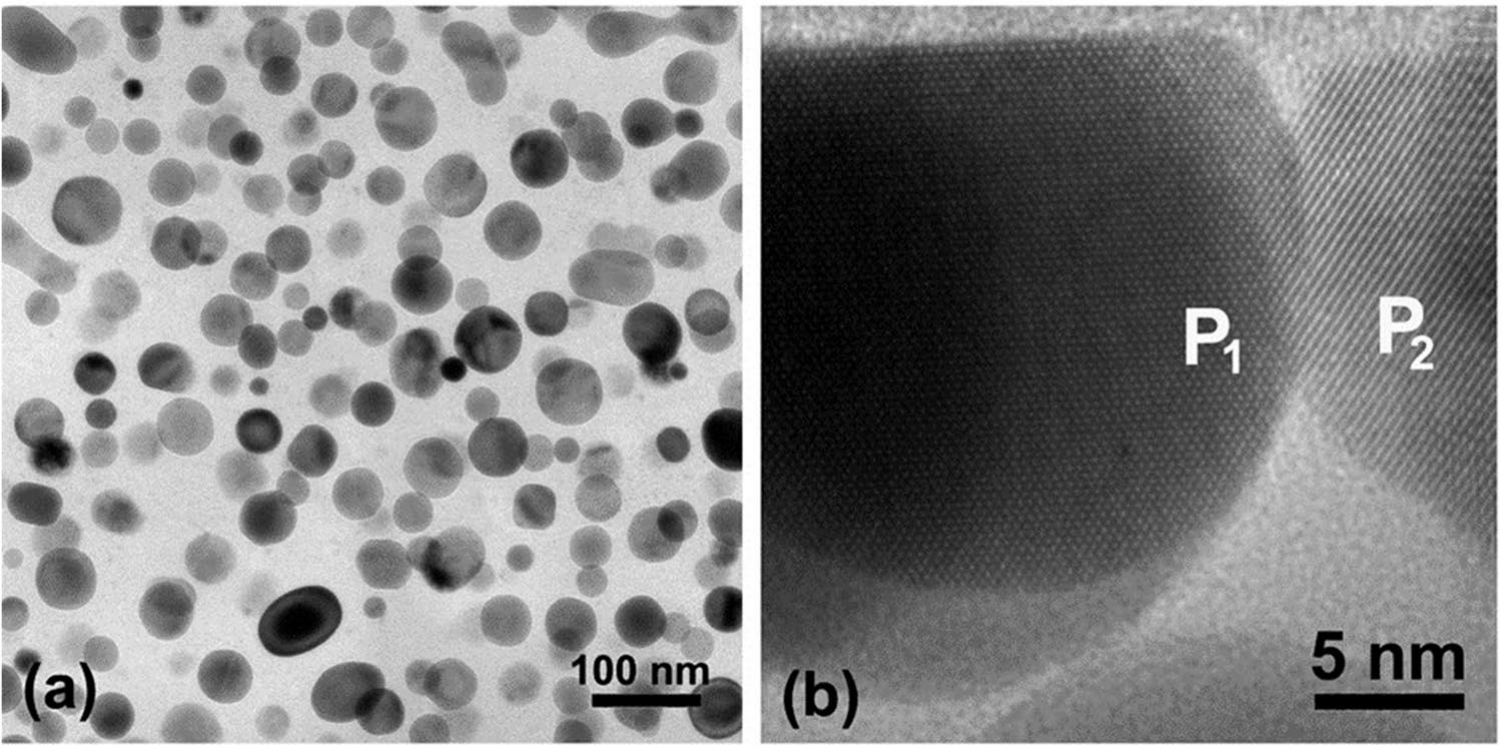
(**a**) Plan-view TEM image and (**b**) cross-section HRTEM image of the top nanocomposite oxidized layer in the annealed Hf_7_B_23_Si_22_C_6_N_40_ film.

As noted earlier, the top oxidized layer in both annealed films exhibited a similar microstructure. Figure 3(a) is a plan-view TEM image of the top nanocomposite oxidized layer of the annealed Hf_7_B_23_Si_22_C_6_N_40_ film showing a large number of HfO_2_ nanoparticles dispersed in a dense SiO_x_-based matrix. The HfO_2_ nanoparticles are mostly spherical with their size varying from ~15 nm to ~80 nm. It can also be seen that in some cases, nanoparticles merge together producing an ellipsoidal shape. HRTEM studies demonstrated the clear matrix has an amorphous structure. The presence of the m-HfO_2_ and o-HfO_2_ nanoparticles in this layer was further verified by HRTEM imaging. For example, the nanoparticle P_1_ in Fig. 3(b) presents two sets of lattice fringes with a spacing of 2.82 Å and 2.63 Å corresponding to (111) and (002) of m-HfO_2_, respectively. Similarly, the nanoparticle P_2_, Fig. 3(b), presents a set of lattice fringes with a spacing of 2.96 Å corresponding to (111) of o- or t-HfO_2_.

Upon annealing of these films, HfO_2_ nanoparticles are first nucleated at the oxidized/bottom layer interface followed by particle coarsening as the top surface is approached, Fig. 2. Subsequently, Si and more than likely B are oxidized forming a protective borosilicate matrix^13^. It has been shown that above 1100 °C, B_2_O_3_ reacts with SiO_2_ forming protective borosilicate glass that fills the pores in the amorphous SiO_2_ structure^39^ resulting in a dense amorphous SiO_x_-based matrix as shown in Fig. 3(a).

#### Microstructure of the bottom layer

**Figure 4:**
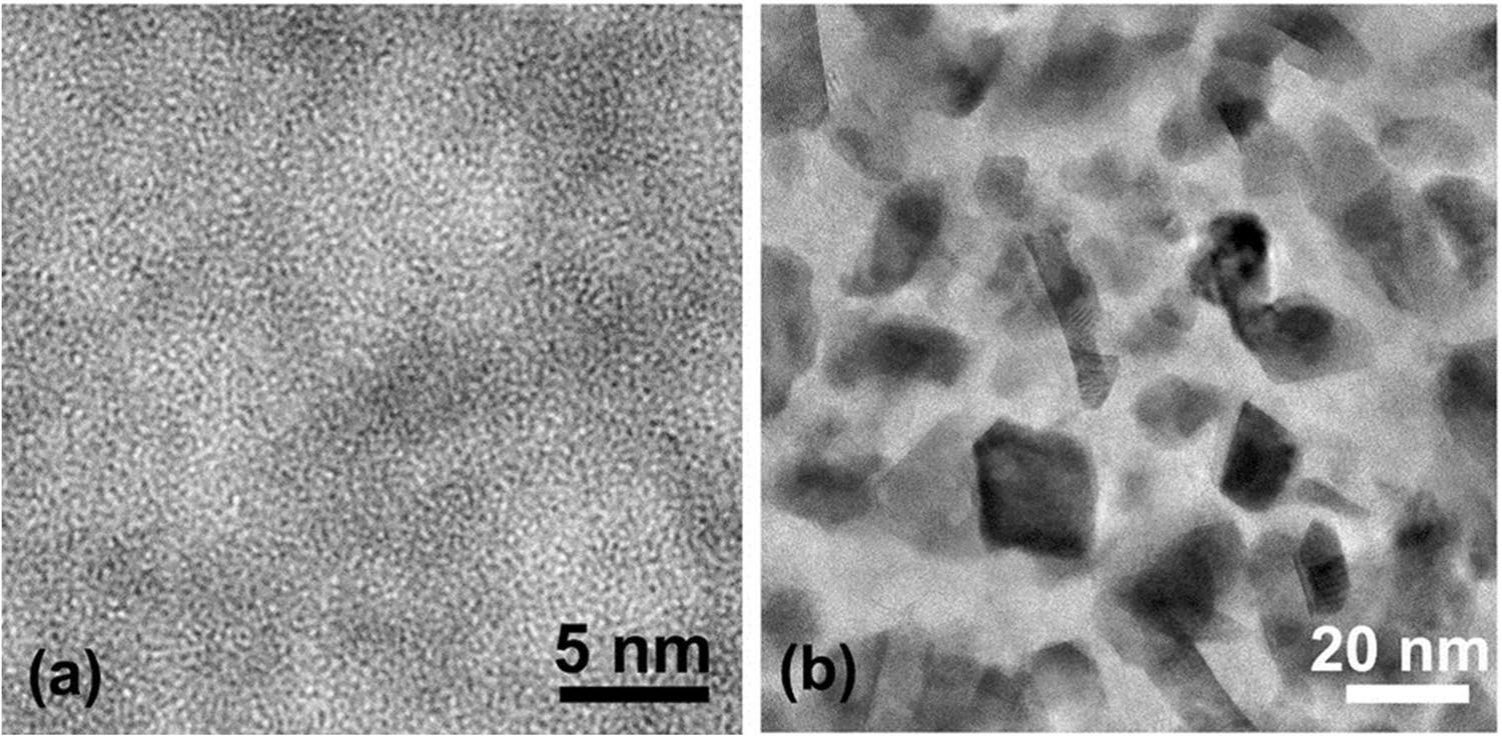
(**a**) HRTEM image of the bottom layer in the annealed Hf_6_B_21_Si_19_C_4_N_47_ film. (**b**) Bright-field TEM image of the bottom layer in the annealed Hf_7_B_23_Si_22_C_6_N_40_ film.

Figure 4(a) is a typical HRTEM image of the bottom layer of the annealed Hf_6_B_21_Si_19_C_4_N_47_ film showing a pure amorphous structure. The regions with darker contrast more than likely correspond to Hf-rich areas and the brighter regions to light element (B, Si, C, N) rich regions. Figure 4(b) is a bright-field TEM image of the bottom layer of the annealed Hf_7_B_23_Si_22_C_6_N_40_ film presenting a quite different microstructure to that shown in Fig. 4(a). Faceted nanoparticles with clear and sharp edges were formed in this bottom film layer. The size of these faceted nanoparticles varies from a few nm up to 20 nm. The nanoparticles are expected to be Hf-rich structures (i.e., HfN, HfB_2_) whereas the boundary regions are rich in light elements. The boundary regions have a very complex structure and HRTEM was intensively used for a comprehensive study of its microstructure as described in the following section.

**Figure 5:**
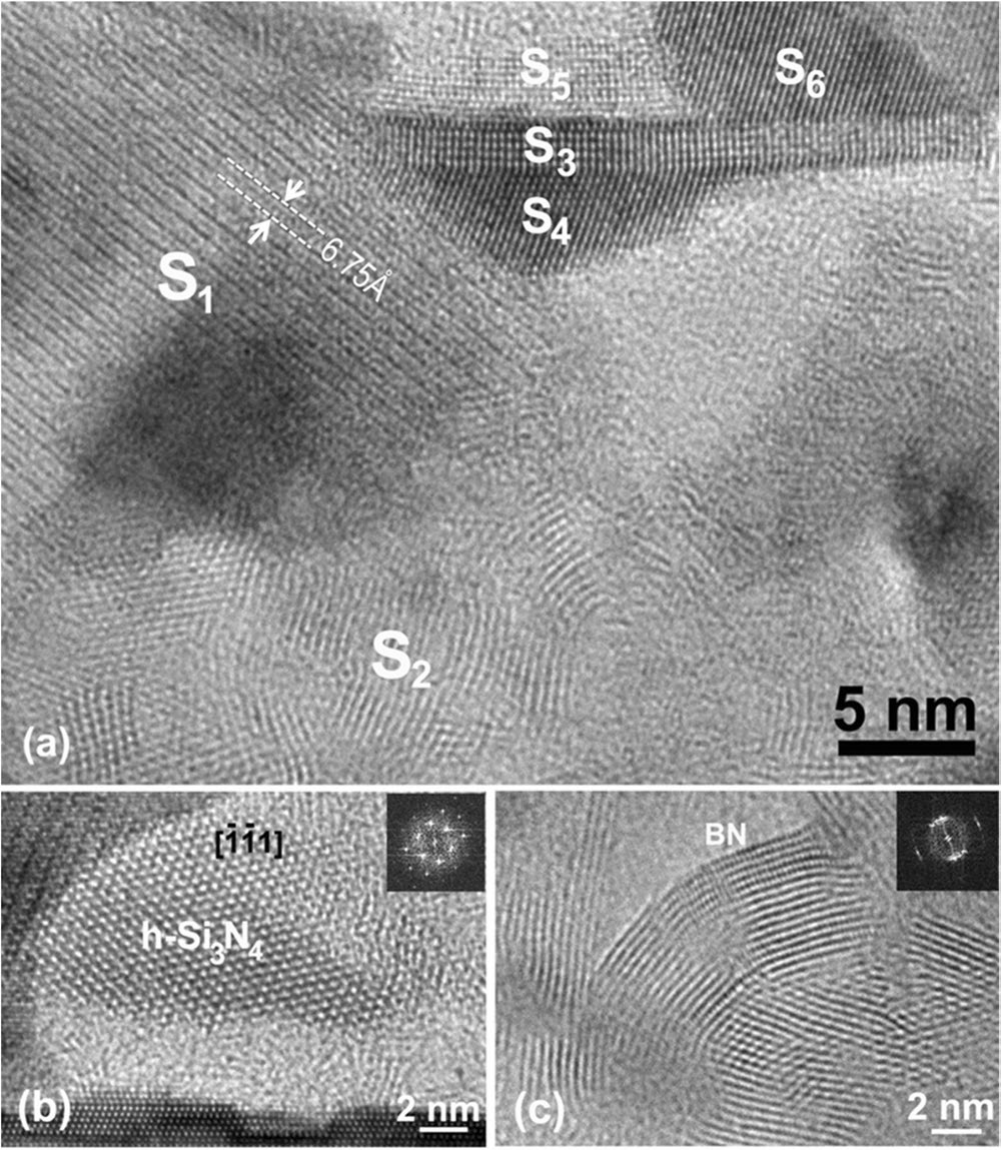
(**a**) HRTEM image taken from the bottom layer in the annealed Hf_7_B_23_Si_22_C_6_N_40_ film, (**b**,**c**) HRTEM images of boundary areas showing lattice images of h-Si_3_N_4_ and BN, respectively.

Figure 5(a) is a HRTEM image taken from the bottom layer of the annealed Hf_7_B_23_Si_22_C_6_N_40_ film presenting a mixture of different phases and structures in this region. The area designated by S_1_ shows lattice fringes with a spacing of 6.75 Å, that is close to the (100) of h-Si_3_N_4_. The presence of h-Si_3_N_4_ in the bottom layer of the annealed Hf_7_B_23_Si_22_C_6_N_40_ film was confirmed by HRTEM images from other regions that show lattice fringes with the electron beam parallel to the zone axis of the h-Si_3_N_4_. One example is shown in Fig. 5(b) presenting a HRTEM image with the electron beam parallel to [$\bar{1}\bar{1}1$] axis of h-Si_3_N_4_ along with the corresponding fast Fourier transformation (FFT). The h-Si_3_N_4_ was found in the light boundary regions shown in Fig. 4(b).

Besides the presence of h-Si_3_N_4_, curved lattice fringes with similar characteristics to an onion structure were frequently observed in the boundary areas, as shown in the S_2_ region in Fig. 5(a). The spacing between the curved lattice fringes is about 3.4 to 3.5 Å, which is very close to the lattice spacing of the (002) of hexagonal BN, which is about 3.33 Å. The nucleation and formation of h-BN has been found in our previous studies of Si-B-C-N film annealed up to 1700 °C in air^28^. The spacing of the curved BN lattice fringes in the latter film was about 3.42 Å, 3% larger than the (002) lattice fringes in the bulk BN, which matches well with the result in the current study. In fact, the somewhat larger lattice spacing can be due to incorporation of C into BN forming a turbostratic B(C)N structure as reported earlier^27,38^. Figure 5(c) presents a HRTEM image and the corresponding FFT from another boundary region showing the curved lattice fringes of BN, in which in addition to the primary lattice fringes of (002)-BN, another set of fringes with a spacing of 2.19 Å corresponding to (100) of BN were observed. This provides solid evidence for the formation of BN in the boundary region in the bottom layer of the annealed Hf_7_B_23_Si_22_C_6_N_40_ film.

The thin, long strip designated by S_3_ in Fig. 5(a), corresponding to a dark area in Fig. 4(b), was identified to be HfB_2_ viewed along its [010] direction. The horizontal lattice fringes have a spacing of 3.45 Å corresponding to (001) HfB_2_ while the vertical fringes have a spacing of 2.71 Å corresponding to (100) HfB_2_. The crystal S_4_ underneath the S_3_ HfB_2_ strip was identified to be fcc HfN viewed along its [110] direction. One set of the {111} planes are parallel to the (001) of HfB_2_. The two regions on the top of the HfB_2_ strip designated as S_5_ and S_6_ are also HfN domains. The lattice fringes in the two regions have a spacing of 2.67 Å, corresponding to (111) of HfN.

Figure 5(a) shows that the HfN nanoparticles S_4_, S_5_, and S_6_ are atomically coherent to the HfB_2_ strip S_3_. After intensive examinations of several other areas, we found that the HfB_2_ crystals were formed in a strip shape and always co-existed with HfN nanoparticles via atomically coherent interfaces. It was interesting to note that even though the HfN nanoparticles can nucleate independently, the HfB_2_ nanostructures were always formed coherently on interfaces with HfN. This tends to indicate that HfB_2_ forms first out of the amorphous structure and its (001) acts as a low energy nucleation site for the HfN phase. Such a sequence of formation is consistent with the lower enthalpy of formation of HfB_2_ compared to HfN^40^.

**Figure 6:**
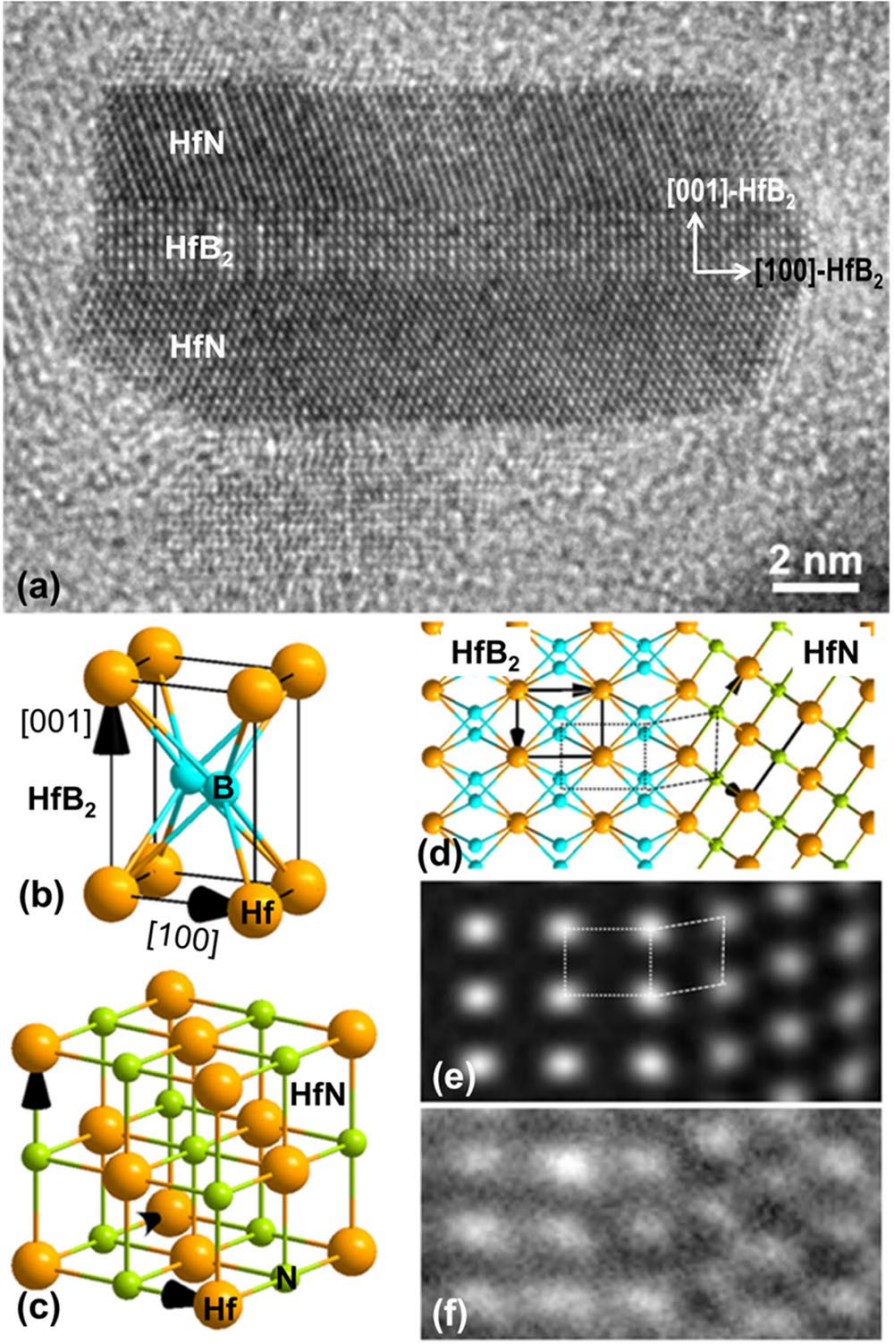
(**a**) HRTEM image of a HfB_2_ and HfN sandwich structure; (**b**,**c**) atomic structure of HfB_2_ and HfN unit cell, respectively; (**d**) projection of coherently joined HfB_2_ and HfN structures along the HfB_2_-[010]; (**e**) a simulated HRTEM image calculated using a defocus of 17 nm and a thickness of 2.3 nm; (**f**) HRTEM image of the HfB_2_/HfN interface.

Figure 6(a) is a representative HRTEM image showing a sandwich structure with a central thin strip of HfB_2_ and two thicker skins of HfN. The central HfB_2_ strip has a thickness of 6 unit dimensions of c-axis (~2 nm) along the vertical direction, i.e., the [001] direction. The long axis of the HfB_2_ strip is along the [100] (horizontal direction) and the [010] is along the normal to the paper. The interfaces between the HfB_2_ core and two HfN skins are the (001) of HfB_2_. They are flat and atomically sharp. The two HfN skins are coherent and atomically joined to the HfB_2_ strip at the (001) of HfB_2_ via a specific orientation relationship:

$${{\rm{HfB}}}_{2} \mbox{-} (001)//\mathrm{HfN} \mbox{-} (111);\,{{\rm{HfB}}}_{2} \mbox{-} [100]//\mathrm{HfN} \mbox{-} [110]\,{\rm{and}}\,{{\rm{HfB}}}_{2} \mbox{-} [010]//\mathrm{HfN} \mbox{-} [\bar{1}01].$$

This coherent joining of the two different structures is not quite surprising and can be understood. HfB_2_ is a close packed hexagonal structure and can be obtained by sequentially packing the close-packed (001) monolayers of Hf (label as “A_Hf_”) and B (label as “B_B_”) along the [001] direction, A_Hf_B_B_A_Hf_B_B_A_Hf_B_B_…, Fig. 6(b). Due to its small size, B atoms can fill the two types of interstices on the top of the Hf monolayer, resulting in the atomic B:Hf ratio of 2:1. HfN is a fcc structure and can be obtained by sequentially packing the closed-packed (111) monolayer of Hf (labelled as A_Hf_, B_Hf_, C_Hf_) and N (labelled as A_N_, B_N_ and C_N_) in the way of A_Hf_B_N_C_Hf_A_N_B_Hf_C_N_A_Hf_B_N_C_Hf_A_N_B_Hf_C_N_…, Fig. 6(c). The Hf monolayers in both HfB_2_ and HfN have the same arrangement. The shortest Hf-Hf bonds (also the major bonds) are 3.21 Å in HfN and 3.14 Å in HfB_2_ (with a small mismatch of ~2%), indicating that the Hf monolayers in HfN and HfB_2_ can be interchangeable. Therefore, it is quite conceivable that HfB_2_ and HfN can coherently join to each other by sharing the Hf monolayer, for example,---A_Hf_B_B_A_Hf_B_B_**A**_**Hf**_B_N_C_Hf_A_N_B_Hf_C_N_---_._ Figure 6(d) shows a projection of such coherently joined HfB_2_ and HfN structure viewed along the direction of HfB_2_-[010]. Figure 6(e) is a simulated HRTEM image of such a structural model calculated using a defocus of 17 nm and a thickness of 2.3 nm. The simulated image matches well with the experimental HRTEM image shown in Fig. 6(f). Actually, the small size of the HfB_2_ strip (~2 nm) and the misfit coherency strain at the HfN/HfB_2_ interface can provide an explanation for the somewhat larger lattice spacing of the (101) HfB_2_ (2.2 Å vs 2.14 Å) observed in the SAED pattern for this phase, Fig. 2(e).

Thus, the present results reveal an interesting crystallization in the bottom layer of the annealed Hf_7_B_23_Si_22_C_6_N_40_ film with formation of HfB_2_ and HfN nanoparticles surrounded by Si_3_N_4_ and BN boundaries. It is more interesting that this crystallization was not observed in the Hf_6_B_21_Si_19_C_4_N_47_ film with higher N content. The results clearly show that even a small difference in the N content can stabilize the amorphous structure at high temperatures.

#### Oxidized/bottom layer interface

**Figure 7:**
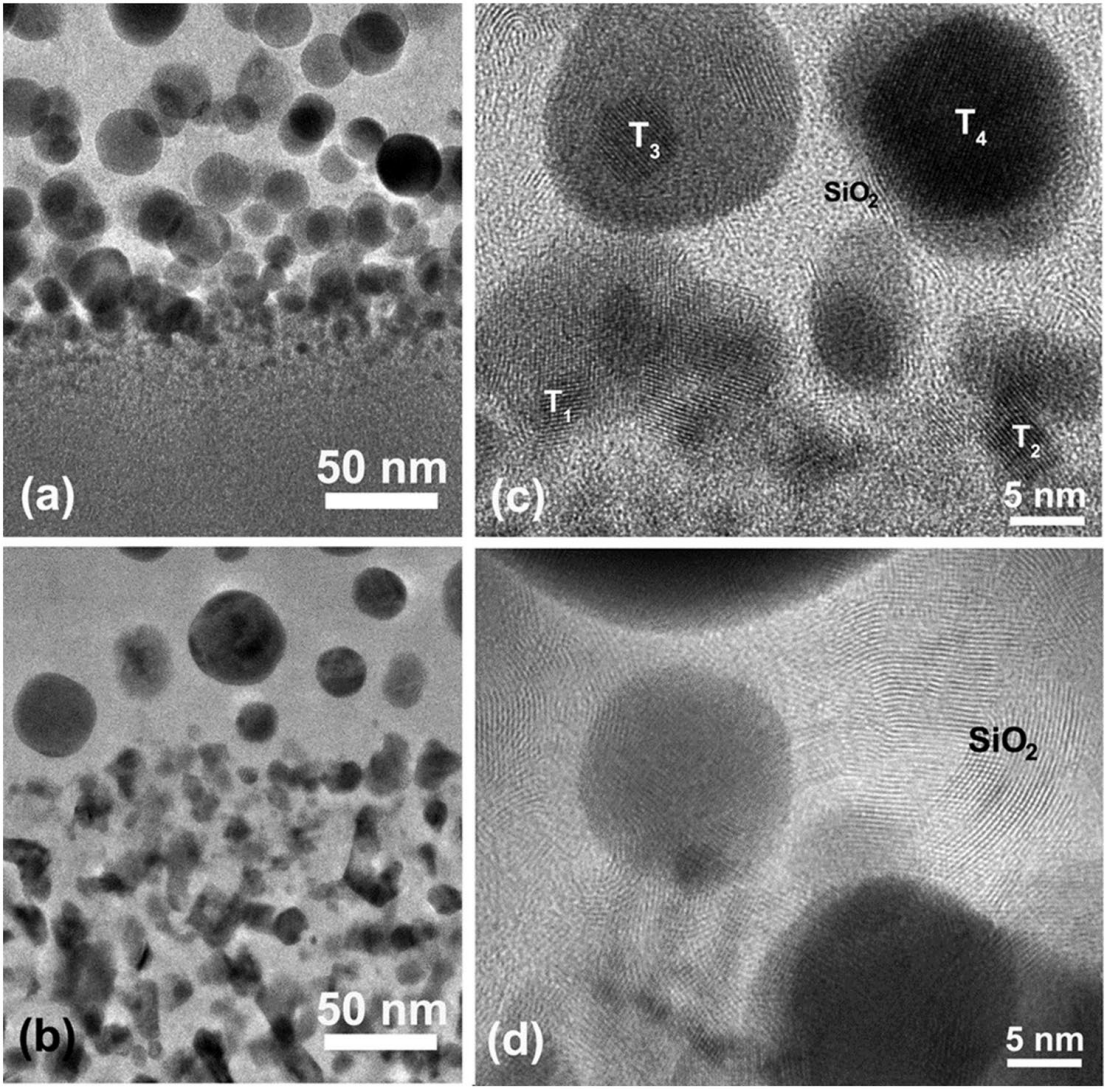
Cross-section TEM and HRTEM image of the oxidized/bottom layer interface in the annealed (**a**,**c**) Hf_6_B_21_Si_19_C_4_N_47_ and (**b**,**d**) Hf_7_B_23_Si_22_C_6_N_40_ film.

The interfaces between the bottom and oxidized layers in both annealed Hf-B-Si-C-N films were further studied using cross-section HRTEM. In the annealed Hf_6_B_21_Si_19_C_4_N_47_ film, densely packed ultra-fine size nanoparticles were nucleated at the interface between the bottom and oxidized layer, Fig. 7(a). These small nanoparticles gradually coarsened into spherical particles (~20 to 30 nm) towards the top surface. Figure 7(c) is a HRTEM image of the interface showing the presence of both m-HfO_2_ (particles T_1_ and T_3_ with (111) lattice fringes and spacing of 2.84 Å) and o-HfO_2_ (particles T_2_ and T_4_ with (101) lattice fringes and spacing of 2.96 Å). The small particles have a size of ~6 nm with an irregular shape (T_1_, T_2_ and T_3_) indicating an early nucleation stage. The larger particle T_4_ that has a size of ~14 nm possesses a spherical shape to minimize surface energy. It was interesting to note that the boundaries between HfO_2_ nanoparticles and matrix were no longer purely amorphous. High temperature, β-quartz SiO_2_ (PDF #11-0252, **a** = 5.002 Å, **c** = 5.454 Å, P3221) formation was observed at the boundary area of the HfO_2_ particles in the interface region, as evidenced by the presence of (101) lattice fringes with a spacing of ~3.4 Å. Thus, the present evidence suggests that as oxygen diffuses and reaches the interface, it reacts with Hf atoms in the amorphous structure and nucleates HfO_2_ nanoparticles. As the amorphous structure is depleted from Hf, Si atoms react with oxygen at the depleted HfO_2_/matrix boundaries forming quartz SiO_2_. In fact, this formation process at the boundaries of the spherical HfO_2_ particles seems consistent with the exhibited (101) SiO_2_ curved lattice fringes instead of straight lines observed in all HRTEM images. It should also be noted that due to their ultrafine scale and curved lattice planes, detection of such a structure by XRD or even SAED is very difficult. It is also important to note that formation of quartz was observed only in the oxidized/bottom layer interface region and not in the upper oxidized layer where more intense oxidation conditions prevail.

Figure 7(b) is a cross-section TEM image of the oxidized/bottom layer interface in the annealed Hf_7_B_23_Si_22_C_6_N_40_ film. As shown earlier, the bottom layer in this film has an entirely different microstructure. During annealing, the amorphous structure was partially crystalized and resulted in the formation of HfB_2_, HfN, Si_3_N_4_, and BN. Thus, as oxygen diffuses in this film and reaches the interface, oxidation of the individual crystalline phases takes place. Due to differences in the oxidation kinetics of the various crystalline phases in the bottom layer of this film, the oxidized/bottom layer interface is not sharp as the one in the Hf_6_B_21_Si_19_C_4_N_47_ film, Fig. 7(a). As shown in Fig. 7(b), a partially oxidized zone develops in the Hf_7_B_23_Si_22_C_6_N_40_ film. Just above the interface, spherical HfO_2_ particles form along with much enriched SiO_2_ crystalline structures, Fig. 7(d), similar to those observed in the Hf_6_B_21_Si_19_C_4_N_47_ film, Fig. 7(a). A closer observation of the image in Fig. 7(d) shows extensive formation of curved quartz lattice fringes that could not have been produced with the same process as that in the Hf_6_B_21_Si_19_C_4_N_47_ film. Quartz formation in this film is attributed to oxidation of pre-existing hexagonal Si_3_N_4_ boundaries around HfN and HfB_2_ nanodomains. Thus, a similar microstructure involving a fine distribution of HfO_2_ nanoparticles surrounded by quartz boundaries developed in the oxidized/bottom layer interface region of both films.

It is important to note that X-ray energy-dispersive spectroscopy analysis showed that both bottom layers in the annealed films are oxygen free. Thus, two entirely different oxidation processes occur in the two films; the first involves oxidation of individual elements out of the amorphous structure and the second, oxidation of nanocrystalline phases (HfB_2_, HfN, Si_3_N_4_, and BN) formed during annealing. In spite of the differences in the aforementioned oxidation process, it should be emphasized that both films exhibit similar microstructures at the oxidized/bottom layer interface and in the top oxidized layer. Furthermore, both films have been found to possess high oxidation resistance with no mass change after annealing up to 1300 °C^32^. It is interesting to note that even though annealing up to 1500 °C produces an oxidized layer of the same thickness (360 nm) in both films, it also results in some small differences in the oxidation behavior that can be attributed to their microstructures. Film Hf_7_B_23_Si_22_C_6_N_40_ exhibits a slow mass gain (less than 10 μg/cm^2^) while film Hf_6_B_21_Si_19_C_4_N_47_ shows negligible mass change^32^. This behavior confirms that both oxidation and volatilization occur simultaneously during annealing. The latter as-deposited film contains more nitrogen and as such, more nitrogen will be released during oxidation resulting in a lower or no mass gain. On the other hand, the former film crystallizes during annealing and therefore, oxygen can penetrate more easily into the film. In this case, oxygen can be present not only in the top 360 nm oxidized layer but also in the partially oxidized interface zone resulting in a mass gain.

Based on the present results, the high temperature oxidation resistance of both films can be attributed to the similar microstructures that develop in the oxidized layer and its interface with the bottom layer in spite of the differences in the oxidation process. In both films, oxidation results in a high density of HfO_2_ nanoparticles within a dense amorphous SiO_x_-based matrix and quartz SiO_2_ formation in front of the base layer. In view of the presence of low thermal conductivity HfO_2_ (0.49–0.95 Wm^−1^K^−1^) nanoparticles, dense SiO_x_-based matrix and high density SiO_2_ nanoquartz (2.5 g/cm^3^), an effective oxygen and thermal diffusion barrier is produced at the interface that can result in high temperature oxidation resistance. It is important to note that the oxidation behavior of the present films produced with the present new deposition process shows significant improvements from previously studied Hf_7_B_23_Si_17_C_4_N_45_ film^13^. Note that the latter film annealed up to 1500 °C exhibited a significantly thicker (544 nm) oxide layer composed of two sublayers. One next to the as-deposited film, composed of a dense population of HfO_2_ nanoparticles (produced by nucleation and growth) and a top, surface sublayer with coarsened and dispersed HfO_2_ nanoparticles (produced by Ostwald ripening). It is interesting that the top sublayer was absent in the oxide layer of the present films that exhibited a much smaller thickness (360–370 nm, more than 30% reduction). Another distinct difference is that a continuous array of HfO_2_ nanoparticles is present at the top surface of the present oxide layers, Fig. 2(a,b). This clearly indicates the high stability of the HfO_2_ nanoparticles in the present microstructures more than likely due to high quality, defect-free films produced by the present deposition process.

Finally, it should be noted that the crystallization observed in the Hf_7_B_23_Si_22_C_6_N_40_ film can actually be utilized as a route to enhance the hardness of the as-deposited film. Hf_7_B_23_Si_22_C_6_N_40_ films annealed in He up to 1300 °C (no oxidation) were found to exhibit a hardness of about 26 GPa due to the evolved nanocrystalline microstructure. Thus, this annealing procedure in an inert environment can be utilized to produce films with high hardness along with excellent high temperature oxidation resistance.

## Summary

Both annealed Hf-B-Si-C-N films exhibit a two-layered structure comprising the original as-deposited film at the bottom and an oxidized layer at the top. In both films, the oxidized layer is composed of m-HfO_2_ and/or o-HfO_2_ embedded in an amorphous SiO_x_-based matrix. The bottom layer in the Hf_6_B_21_Si_19_C_4_N_47_ film remains amorphous while that in the Hf_7_B_23_Si_22_C_6_N_40_ film is partially crystallized exhibiting a nanocomposite structure of HfB_2_ and HfN nanoparticles surrounded by h-Si_3_N_4_ and h-BN boundary phases. The HfB_2_ and HfN nanoparticles found to form a sandwich structure with a HfB_2_ strip core being atomically coherent to HfN skins via a (111) Hf monolayer. The oxidized/bottom layer interface of the Hf_6_B_21_Si_19_C_4_N_47_ film is characterized by a high density of HfO_2_ nuclei surrounded by SiO_2_ quartz boundaries resulting from the oxidation of the amorphous structure. The interface of the Hf_7_B_23_Si_22_C_6_N_40_ film shows a similar HfO_2_/SiO_2_ quartz boundary microstructure that is a result of oxidation of the crystalline phases (HfN, HfB_2_ and h-Si_3_N_4_) produced during annealing. The high temperature oxidation resistance of these films is attributed to the particular microstructure involving formation of HfO_2_ nanoparticles in a SiO_x_-based matrix along with quartz formation at the oxidized/bottom layer interface that acts as a barrier for oxygen and thermal diffusion.

## Data Availability

All experimental deposition conditions and characterization procedures, methods and data are provided in the text. Any clarifications will be available by contacting the corresponding author.
